# Rosetta FlexPepDock *ab-initio*: Simultaneous Folding, Docking and Refinement of Peptides onto Their Receptors

**DOI:** 10.1371/journal.pone.0018934

**Published:** 2011-04-29

**Authors:** Barak Raveh, Nir London, Lior Zimmerman, Ora Schueler-Furman

**Affiliations:** 1 Department of Microbiology and Molecular Genetics, Hadassah Medical School, Institute for Medical Research Israel-Canada, The Hebrew University, Jerusalem, Israel; 2 The Blavatnik School of Computer Science, Tel-Aviv University, Tel-Aviv, Israel; University of South Florida College of Medicine, United States of America

## Abstract

Flexible peptides that fold upon binding to another protein molecule mediate a large number of regulatory interactions in the living cell and may provide highly specific recognition modules. We present Rosetta FlexPepDock *ab-initio*, a protocol for simultaneous docking and *de-novo* folding of peptides, starting from an approximate specification of the peptide binding site. Using the Rosetta fragments library and a coarse-grained structural representation of the peptide and the receptor, FlexPepDock *ab-initio* samples efficiently and simultaneously the space of possible peptide backbone conformations and rigid-body orientations over the receptor surface of a given binding site. The subsequent all-atom refinement of the coarse-grained models includes full side-chain modeling of both the receptor and the peptide, resulting in high-resolution models in which key side-chain interactions are recapitulated. The protocol was applied to a benchmark in which peptides were modeled over receptors in either their bound backbone conformations or in their free, unbound form. Near-native peptide conformations were identified in 18/26 of the bound cases and 7/14 of the unbound cases. The protocol performs well on peptides from various classes of secondary structures, including coiled peptides with unusual turns and kinks. The results presented here significantly extend the scope of state-of-the-art methods for high-resolution peptide modeling, which can now be applied to a wide variety of peptide-protein interactions where no prior information about the peptide backbone conformation is available, enabling detailed structure-based studies and manipulation of those interactions.

## Introduction

Peptide-mediated interactions with globular proteins play a prominent role in signaling and regulatory networks of the living cell [1], [2]. It has been estimated that between 15%-40% of all protein-protein interactions are mediated by flexible peptide that fold upon binding to a globular receptor [2]. These peptides often form a modular binding motif, which can be embedded in intrinsically unstructured protein regions and within flexible loops, in order to confer desired interactions [2], [3], [4], [5].

Due to their cardinal role in regulatory mechanisms (e.g. [6]), flexible peptides are implicated in human disease and cancer [1], and therefore provide attractive leads for the design of inhibitory peptides and small molecule drugs [7], [8], [9], [10], [11], [12]. A large-scale *in-silico* survey that we conducted recently suggests that peptides derived from globular proteins often have the potential to disrupt interactions of their origin domains by competitive inhibition [13], as was previously shown in experiment in several physiological interactions [14], [15]. Hence, peptide molecules and their derivates hold great potential for targeted modulation of the cellular network of protein interactions.

Available structural models of peptide-protein interactions obtained by X-ray and NMR experiments have contributed significantly to our understanding of the mechanisms underlying key cellular interactions [9], [16], [17], [18], and enabled the structure-based redesign of both the peptide and receptor sequence at the binding site to inhibit specific cellular interactions altogether [10], [11]. However, the number of available models solved in experiment represents only a small fraction of known peptide-protein interactions, while high-throughput methods for screening of peptide libraries such as peptide arrays [19] combinatorial phage-display [20], [21], [22] and yeast surface display [23], [24], [25] continue to produce an ever increasing flux of data about new peptide-mediated interactions whose structural basis is mostly poorly understood.

We have recently introduced Rosetta FlexPepDock [26], a protocol for the refinement of coarse models of peptide-protein complex structures. We benchmarked FlexPepDock thoroughly to define an effective basin of attraction of 5.5 Å RMSD from which the protocol can reliably recover near native peptide conformations. This covers a wide range of real world biological problems, for which an initial approximate structure is available. Indeed, we have used this protocol to model the structure of different peptide-protein interactions and to learn about their functional role (e.g. [27], [28], [29]). However, refinement is effective only if the approximate peptide backbone conformation within the receptor-binding site is given. Other methods dedicated to peptide docking have recently been developed but seem to be rather local as well [30], [31], [32], or restricted to very short peptides [33], [34].

In this study, we introduce Rosetta FlexPepDock *ab-initio*, which is designed to address the subset of problems where the approximate location of the peptide binding site is known, but no information about the peptide backbone conformation is available. The approximate binding site of the peptide can often be obtained from cross-linking experiments, mutational analysis, NMR shifts or any other experimental evidence [35], [36], and from computational predictions of increasing quality [37]. FlexPepDock *ab-initio* borrows from existing Rosetta protocols, and attempts to ‘fold’ the peptide at the binding site, using fragment-based sampling to detect the overall conformation of the peptide in a reduced representation space (centroid mode) [38], coupled with efficient scanning of peptide orientations over the protein surface. This step is then followed by all-atom refinement of the peptide-protein conformation with fine backbone modeling and side-chain placement, based on the Rosetta FlexPepDock refinement protocol [26].

In the following sections, we present the Rosetta FlexPepDock *ab-initio* protocol, its usage and workflow and its performance on a selected benchmark of peptide-protein complexes. We anticipate that the new protocol will significantly contribute to the study of peptide-protein interactions, both for the purpose of basic research and for the increasing use of peptides for pharmaceutical applications.

## Methods

**Figure 1:**
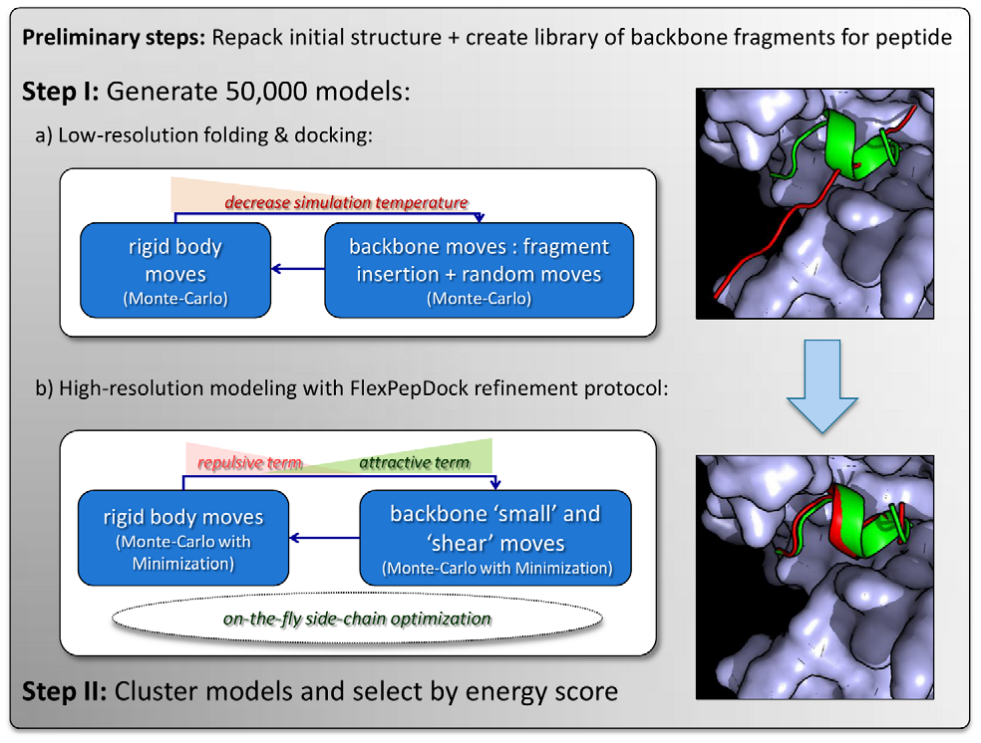
General outline of the Rosetta FlexPepDock*ab-initio* protocol. See Text for more details.

Fig. 1 shows a schematic view of the FlexPepDock *ab-initio* protocol. Each step is described in more detail below.

### Preliminary steps prior to running the protocol

#### Input model

The input to the protocol is an initial model of the peptide-protein complex. It is assumed that the receptor backbone is approximately correct, and that the peptide is initially positioned close to the correct binding site, albeit with arbitrary backbone conformation (the present study is based on results starting from extended peptide backbone conformations superimposed on a randomly selected anchor residue, but the protocol is designed to work from any arbitrary peptide starting conformation; see below and Table S3). Initial side-chain coordinates (such as the crystallographic side-chains of an unbound receptor) can be optionally provided as part of the input model, but are not required. In our analysis, we discarded the input side-chains from native complexes in the bound runs, but kept the side-chains from the free receptor structures in the unbound runs.

#### Preparation of fragment library for peptide

For efficient sampling of the peptide backbone, we generate a library of trimer, pentamer and nonamer backbone fragments, which are extracted from solved protein structures in the Protein Data Bank (PDB at www.pdb.org [39]), using the protocol described in this issue by Gront *et al*. The library is constructed based on sequence similarity to the query peptide and on the secondary structure predicted for the peptide by PSIPRED [40], resulting in 500 fragments from each category of secondary structure type, *i.e.*, α helix, extended β strand and coiled-coil loop (with a total of 1,500 fragments for a given query peptide). We note that although PSIPRED was not optimized for peptides, the resulting fragment libraries showed in practice good coverage of the peptide conformational space (in concordance with a recent report by Vanhee *et al.* that most peptide-protein interactions can be represented by fragment sets derived from single, non-related monomer structures [17]). We also note that fragments used in this analysis were not taken from the native peptide structure or any of its homologues (in fact, only fragments from globular monomer proteins were considered).

See the FlexPepDockAbInitio protocol capture archive:

scripts/prep_abinitio.sh pdb-id

#### Prepacking step -remove internal clashes in receptor

A first preliminary step in our protocol involves the packing of the side-chains in each monomer to remove internal clashes that are not related to inter-molecular interactions, as described in Raveh *et al.* [26].

The pre-packing stage guarantees a uniform conformational background in non-interface regions, prior to molecular docking [41]. We refer to the pre-packed input structure as the *starting structure*.

See the FlexPepDockAbInitio protocol capture archive:

$PATH_TO_EXE/FlexPepDocking.linuxgccrelease -database $PATH_TO_DB –s start.pdb -native native.pdb -ex1 -ex2aro -use_input_sc -unboundrot native.pdb -flexpep_prepack -nstruct 1

### Step I: generation of models

We generate a large number of models beginning from the starting structure by repeating the procedure described below over multiple independent runs (which can be performed in parallel on a CPU cluster). In the present study we generated 50,000 models from each starting structure. More models can improve sampling and results further.

#### Step Ia – fast low-resolution modeling

In a first step, the peptide is folded and docked over the surface of the receptor protein using a low-resolution representation of the complex, in which the side-chains are represented as unified spheres (Rosetta centroid mode [38]). The peptide is alternately folded and docked for 10 outer cycles. Each such outer-cycle consists of two internal Monte-Carlo simulations. The temperature term of the Metropolis criterion in the internal simulations is gradually decreased from 2.0 in the first outer cycle to 0.6 (arbitrary units) in the last outer cycle, such that large perturbations are favored in the first rounds. The inner simulations consist of: *(1) Optimization of the rigid-body orientation:* The peptide rigid-body orientation is optimized by a Monte-Carlo simulation consisting of 50 random rigid-body transformations (translational magnitude of 1 Å and rotational magnitude of 10° on average in each step). *(2) Optimization of the peptide-backbone:* The *ab initio* sampling protocol of the peptide backbone is performed based on moves described in detail in Rohl *et al.* [38]. In short, the peptide backbone conformation is perturbed over 50 random Monte-Carlo moves while the peptide rigid body orientation remains fixed. Each move is selected randomly. In 60% of the moves, the ϕ/ψ torsion angles of random residues are perturbed using the so-called ‘small’ and ‘shear’ random moves described in Rohl *et al.* [38], to random magnitude, corresponding for the ‘small’ moves to random changes in ϕ/ψ angles. In the shear moves, the ϕ angle is rotated with equal magnitude but opposite direction relative to the preceding ψ angle, thereby reducing the perturbation to the rest of the chain. For both moves, perturbations to non-favorable Ramachandran angles are discriminated against, using a Metropolis criterion. In the remaining 40% of the moves, a trimer (30% of cases), pentamer (7.5% of cases) or a nonamer (2.5% of cases; for peptides with nine residues or more) fragment from the fragment library is inserted in a random position within the peptide.

#### Step Ib – refinement of low-resolution model

The low-resolution modeling step results in a coarse-grained model of the peptide-protein complex. This model is further optimized using high-resolution refinement with the Rosetta FlexPepDock refinement protocol [26]. This protocol was shown to be effective when the initial peptide conformation lies up to 5.5 Å from the native conformation. In brief, it consists of alternating optimization of the peptide rigid-body and backbone and orientation using the Monte-Carlo with Minimization approach [42] and a set of small-scale perturbations. To allow significant perturbations within the binding pocket while preventing the peptide and protein to separate during energy minimization, the refinement step begins with decreased and increased weights for the repulsive and attractive van der Waals term in the energy function, respectively. During refinement, these terms are gradually ramped back towards their original values.

See the FlexPepDockAbInitio protocol capture archive:

$PATH_TO_EXE/FlexPepDocking.linuxgccrelease -database $PATH_TO_DB

#io flags:

-s start.ppk.pdb

-native native.pdb

-out:file:silent_struct_type binary

-out:file:silentdecoys.silent

-scorefile score.sc

#If using multiple processes and no silent file:

#-multiple_processes_writing_to_one_directory

#number of structures to produce

#for demo:

-nstruct 5

#for production run:

#-nstruct 50000

#flexpepdock flags:

-rbMCM

-torsionsMCM

-flexPepDocking:lowres_abinitio

-flexPepDocking:flexpep_score_only

#packing flags

-ex1

-ex2aro

-use_input_sc

-unboundrot native.pdb

#fragment picker flags:

-frag3 frags/frags.3mers.offset

-frag9 frags/frags.9mers.offset

-flexPepDocking:frag5 frags/frags.5mers. offset

-flexPepDocking:frag5_weight 0.25

-flexPepDocking:frag9_weight 0.1

### Step II: selection of models

In addition to sampling the conformational energy landscape efficiently, the challenge of modeling includes also the selection of the correct model among all the created models, which can be illustrated by the notion of finding a needle in a haystack. In order to do so, we first cluster our top-scoring models, and subsequently select top-scoring clusters as a model for the interaction. The top scoring 500 models are clustered using the Rosetta Cluster application, as described in Gray *et al.* [41], with a cluster radius cutoff of 2 Å peptide backbone atom RMSD. From each cluster, a representative model is subsequently selected according to the best energy score. The clusters are then ranked according to the energy of their representative models.

See the FlexPepDockAbInitio protocol capture archive:

scripts/clustering/cluster.sh pdb-id topXrms-radius scorefile reference-pdb models-silent-file score-type

### Benchmarking of protocol

#### Datasets of peptide–protein interactions

Since the FlexPepDock *ab-initio* simulations are time intensive, we selected a small but representative subset of complexes on which to assess the protocol. The bound dataset used in this study includes 26 peptide–protein complex structures (Table S1) chosen from the peptiDB dataset [9], a non-redundant set of high-resolution peptide–protein complex structures (below 70% sequence identity between receptor proteins; structures solved at resolution of 2 Å or better). These peptide-protein complexes represent a wide range of biological contexts. Besides a few interactions with a known and well-defined motif (PDB ids: 1SSH, 1W9E, 1Z90 and 2P1K), the interactions in this dataset were selected randomly. The length of peptides in the dataset varies between 5 and 13 amino acids, with up to 52 rotatable bonds. More details about these complexes can be found in Table S1.

#### Unbound dataset

For 14 out of the 26 complexes tested in this study, a high resolution (<2 Å) free receptor structure has been solved (or that of a protein with >90% sequence identity). The unbound structures were also extracted from the peptiDB dataset [9] and their interface residues were superimposed onto their bound counterparts as described in London *et al.* [9], to evaluate the difference between the free and bound receptor (Table S1). We note that in our benchmarking analysis, we discarded the input side-chains from native complexes only for docking to bound receptor structures, but not to free receptor structures (as this information is available in a real world scenario): including side-chains of unbound receptors was shown in our previous docking studies to improve protocol performance [26], [43].

#### Measure of success

We define a docking model as *near-native* if the interface backbone atoms of the predicted peptide conformation deviate by ≤2 Å RMSD. For a docking simulation of a given interaction, we define *successful sampling* as the cases where a near-native model is sampled, and *successful ranking* as the cases where a near-native model is ranked among the ten lowest-energy clusters.

#### Extending the peptide from a random anchor

For testing the protocol starting from an extended peptide conformation, a random peptide position was selected as an anchor (see Table S1 for details), and its coordinates were extracted from the native complex. The peptide's ϕ/ψ angles were then set to canonical ideal extended conformation (+135°/−135°, respectively), from which the docking simulations were initiated. We note that the random anchor was not specified, and the peptide was completely free to move during the simulation.

#### Random rigid-body perturbations

In order to evaluate the robustness of FlexPepDock *ab-initio*, we repeated the run from a different orientation, created by perturbation of the rigid-body orientation of the extended peptide by random Gaussian translations and rotations of magnitudes 3 Å and 30°, respectively. Docking experiments were then initiated from the perturbed extended conformation.

#### Rosetta Revision

The protocol and tests described in this manuscript follow the FlexPepDock protocol as implemented within revision 39664 of the Rosetta repository.

#### Running time

A single simulation takes 2–4 minutes on a single CPU over an AMD Sun cluster, depending on the size of the receptor protein. Generation of the entire 50,000 models for a single run takes approximately 24 hours on a cluster of 120 processors.

### Rosetta infrastructure

As for the original *FlexPepDock* refinement protocol, the *FlexPepDock ab-initio *protocol is fully implemented within the Rosetta modeling framework [44]. Rosetta provides well-calibrated energy functions, efficient energy calculations and a battery of established conformational sampling protocols. In particular, we use the Rosetta library of protein fragments extracted from solved protein structures [38], the Monte-Carlo sampling with Energy Minimization first proposed by Li andScheraga [42], the Rosetta side-chain repacking protocol [45] and the Dunbrackrotamer library [46]. For energy scoring, we use a modified version of the Rosetta full-atom energy function (Rosetta score12 [38], [45], see below) and the coarse-grained energy function, which employs a unified spheres side-chains model (Rosetta centroid score4 [38]).

### Energy function used for model selection

In our original FlexPepDock refinement study [26] we used the standard Rosetta scoring function - score12 to rank and select the top-scoring models. However, several previous docking studies with Rosetta have indicated that the interface score, *i.e.* the energy score across the interface (where only atom-atom contacts between the partners are included in the energy evaluation) provides better estimates by removing effects outside from the actual interface. In addition, studies in our group on the prediction of binding specificity have shown that the score of the peptide (*i.e.* the internal peptide energy together with the interface energy) provides the best estimate of binding (unpublished data). We therefore investigated different energy terms for their ability to identify the near-native models among the set of created models. The total energy, interface energy, peptide energy, and a weighted sum of all were assessed (see Table S2).

See the FlexPepDockAbInitio protocol capture archive:

scripts/scoring/rescore.sh score.sc

### Guide to installing and using the protocol

A detailed guide to the protocol with examples can be found as a Protocol Capture archive named FlexPepDock Ab Initio.

## Results and Discussion

### I. General outline of Rosetta FlexPepDock *ab-initio* protocol

The main components of our protocol for simultaneous *ab-initio* folding and docking of flexible peptides are outlined in Fig. 1. In short, after removing internal clashes of the receptor structure (by repacking), we place the peptide into the binding site (here as an extended conformation), and compile a corresponding library of peptide backbone fragments. We then apply FlexPepDock *ab-initio*, starting with low-resolution optimization of the peptide backbone conformation and its rigid-body orientation, followed by high-resolution refinement with full flexibility for all peptide and receptor side-chains. The high-resolution step corresponds to our previously developed Rosetta FlexPepDock refinement protocol [26]. More details can be found in the *Methods* section.

### II. Large-scale assessment of protocol performance

**Figure 2:**
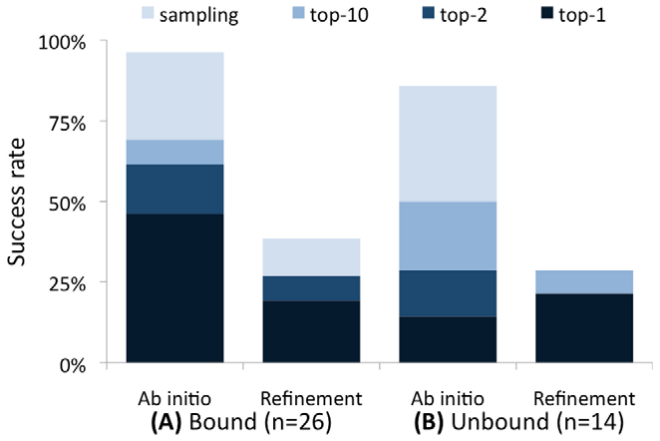
Summary of performance of FlexPepDock *ab-initio* on a benchmark of peptide-protein complexes. The FlexPepDock *ab-initio* protocol (**Ab initio runs**) samples a near-native structure in most of the Bound (**A**) and Unbound (**B**) simulations (height of bars), and in a significant fraction of the complexes a near-native structure is identified by the top-ranking cluster, or among the top-2 or top-10 (shaded parts of the bar; models clustered according to 2 Å peptide backbone RMSD cutoff). This significantly increases the scope FlexPepDock when compared to the original refinement protocol (**Refinement runs**), for both the Bound and Unbound simulations.

**Figure 3:**
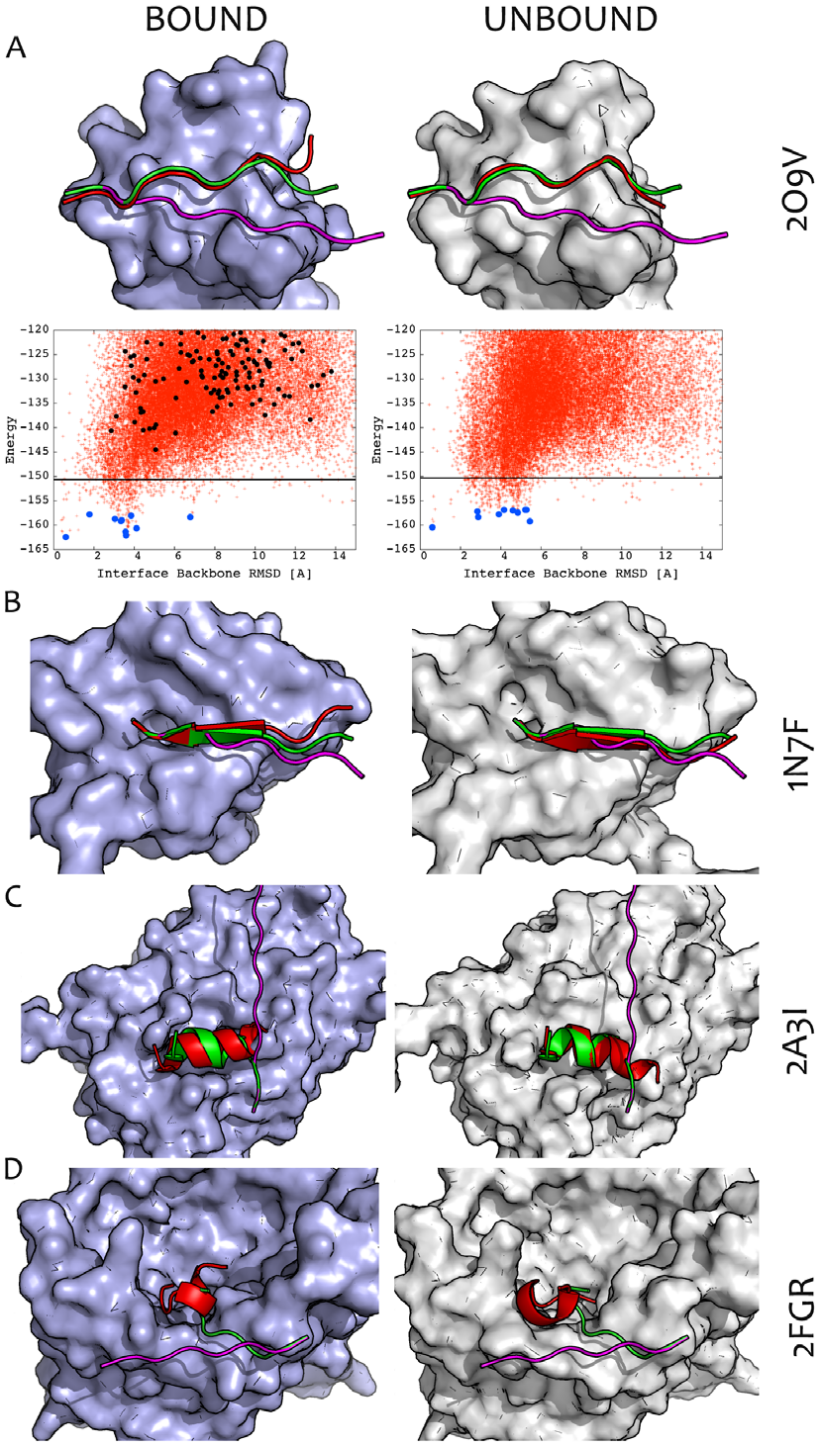
Examples of models created by the *ab-initio* FlexPepDock protocol. Bound (left panel) and Unbound (right panel) docking runs are shown for three successful and one failed simulation (see Table 1 for the full dataset): (**A**) 2O9V (Ponsin SH3 domain -paxillin proline rich region [51]); (**B**) 1N7F (GRIP1 PDZ6-liprin α c-terminal peptide complex [52]); (**C**) 2A3I (Mineralocorticoid ligand receptor domain - LXXLL motif of steroid receptor coactivator-1 (SRC1-4) [53]); and (**D**) 2FGR (Omp32-PAP peptide [54]). For 2O9V, 1N7F and 2A3I, a near-native model was ranked among the top 10 clusters for both the bound and unbound cases (the bound run of 2A3I is a borderline case, with 2.1A backbone interface RMSD). For each complex, the receptor is shown in either lavender or gray shade, for the Bound or Unbound form, respectively. The cartoonre presentation shows the peptide starting orientation (magenta), final model (red), and native structure (green). The corresponding energy landscape plots are shown for (A) (see Fig. S1 for additional plots for all examples). Each model created by FlexPepDock *ab-initio* is plotted as a red cross according to its peptide interface backbone RMSD (x-axis) and its energy score (y-axis; reweighted score; see Methods). The top 10 lowest energy clusters created from the top 500 scoring models are shown as blue circles. The black line indicates the maximal energy of the top 500 models. The energy plots show a sub-Ångstrom lowest-energy cluster for both Bound and Unbound simulations. The black circles in the left panel show results from the previous FlexPepDock refinement protocol [26], demonstrating how increased sampling allows the detection of conformations that have better energy scores and are also more similar to the native structure.

We assessed the ability of FlexPepDock *ab-initio* to sample and identify the correct conformation of peptides on a benchmark of 26 different peptide-protein interactions extracted from the PeptiDB dataset [9] (Table S1 and Methods). We created n = 50,000 conformations, starting from an extended peptide within the binding site. The resulting models were clustered and the clusters were ranked based on a reweighted version of the Rosetta generic full-atom energy score, in which interface and peptide residuesare given additional weight, and which improves the performance of the protocol compared to the standard Rosetta score (see Table S2 and Methods). Finally, we assessed how many runs succeeded to identify near-native models (defined as models with ≤2 Å peptide interface backbone RMSD in the top 10 scoring clusters; see Methods). The performance of FlexPepDock *ab-initio* is summarized in Table 1 (bound docking), Table 2 (unbound docking) and Fig. 2, and specific examples are presented in Fig. 3.

**Table 1:** Benchmark of FlexPepDock *ab-initio*.

(A) Bound PDB id	Sec str	Start backbone-RMSD∫	Start ϕ/ψ RMSD∫	Best backbone- iRMSD†	Top-10 backbone-iRMSD‡	Rank of first near-native cluster	Hexamer *all-atom* top-10*
**1NVR^a^**	β + C	1.2 Å	23°	0.2 Å	**0.5 Å**	**1**	0.9 Å^b^
**2FMF^c^**	A	16.1 Å	131°	0.4 Å	**0.5 Å**	**7**	0.6 Å
**2O9V^c^**	C	5.5 Å	49°	0.4 Å	**0.6 Å**	**1**	0.4 Å
2P1K	β + C	6.1 Å	61°	0.5 Å	**0.6 Å**	**1**	0.6 Å
**1RXZ**	β + C	8.9 Å	80°	0.7 Å	**0.7 Å**	**1**	0.9 Å
2R7G	α + C	11.2 Å	106°	0.8 Å	**0.8 Å**	**7**	1.1 Å
**1AWR**	C	5.3 Å	57°	0.8 Å	**0.9 Å**	**1**	1.6 Å
2FNT	C	1.4 Å	47°	0.4 Å	**1.0 Å**	**1**	2.0 Å
3D1E	C	1.6 Å	67°	0.7 Å	**1.1 Å**	**1**	1.3 Å
2B1Z	A	8.9 Å	124°	0.4 Å	**1.2 Å**	**1**	2.2 Å
**1T7R**	A	13.4 Å	128°	0.7 Å	**1.2 Å**	**3**	0.8 Å
**1N7F^c^**	β + C	3.8 Å	52°	0.3 Å	**1.4 Å**	**1**	0.8 Å
1ER8	C	6.0 Å	52°	0.8 Å	**1.4 Å**	**1**	1.9 Å
**1W9E**	β + C	1.2 Å	39°	0.5 Å	**1.5 Å**	**1**	2.7 Å^b^
**2VJ0**	C	5.3 Å	72°	1.2 Å	**1.5 Å**	**1**	1.4 Å
1Z9O	C	6.9 Å	56°	1.6 Å	**1.6 Å**	**2**	1.4 Å
**2P54**	α + C	13.0 Å	120°	0.8 Å	**1.8 Å**	**2**	2.2 Å
1NLN	β + C	11.7 Å	27°	0.7 Å	**2.0 Å**	**5**	1.7 Å
**2A3I^c^**	α + C	17.3 Å	115°	0.7 Å	**2.1 Å**	**314**	2.2 Å
**1SSH**	C	8.4 Å	50°	0.9 Å	**3.1 Å**	**51**	2.7 Å
2J6F	C	7.4 Å	44°	1.7 Å	**5.7 Å**	**>500**	7.2 Å
1KL3	α + C	9.4 Å	93°	0.9 Å	**5.8 Å**	**>500**	6.5 Å
1TW6	C	3.3 Å	46°	1.8 Å	**5.9 Å**	**>500**	6.5 Å
**2C3I**	C	13.0 Å	70°	1.5 Å	**6.0 Å**	**308**	4.7 Å
**2FGR^c^**	C	7.3 Å	79°	1.3 Å	**8.6 Å**	**>500**	8.6 Å
1QKZ	C	13.51Å	78°	3.1 Å	**8.8 Å**	**None**	4.5 Å

Performance of peptide modeling onto Bound (A) and Unbound (B) protein receptor structures.

The proteins in the benchmark are detailed in Table S1 (See Methods for more details).

∫ The start bb-RMSD and start ϕ/ψ RMSD refer to the distance between the initial peptide conformation at the beginning of the simulation and the native peptide backbone.

† The best peptide backbone interface-RMSD among all 50,000 sampled models.

‡ The best peptide backbone interface-RMSD within the ten models representing the top clusters.

*The best *all-atom* RMSD of any partial hexamer of the peptide, among the ten top ranking models.

a PDBids in italics indicate complexes modeled also on the free receptor conformation (see Table 2).

b Pentamer.

c Complexes described in accompanying Figures.

**Table 2:** Benchmark of FlexPepDock *ab-initio*.

Unbound PDB id	Unbound receptor Cα- iRMSD	Sec str	Start backbone-RMSD^∫^	Start ϕ/ψ-RMSD^∫^	Best backbone- iRMSD†	Top-10 backbone-iRMSD‡	Rank of first near-native cluster	Hexamer*all-atom* top-10^*^
1NVR	0.3 Å	β + C	1.2 Å	23°	0.2 Å	**0.4 Å**	**2**	1.1 Å^b^
2O9V^c^	0.3 Å	C	5.5 Å	49°	0.4 Å	**0.6 Å**	**1**	0.6 Å
1N7F^c^	0.4 Å	β + C	3.8 Å	52°	0.8 Å	**1.0 Å**	**9**	1.2 Å
2VJ0	0.3 Å	C	5.3 Å	72°	1.2 Å	**1.4 Å**	**8**	1.8 Å
1AWR	0.3 Å	C	5.3 Å	57°	0.9 Å	**1.4 Å**	**2**	2.0 Å
2A3I^c^	0.3 Å	α + C	17.3 Å	115°	0.7 Å	**1.5 Å**	**10**	1.7 Å
1W9E	0.6 Å	β + C	1.2 Å	39°	0.7 Å	**1.9 Å**	**1**	2.8 Å^b^
1T7R	0.4 Å	α	13.4 Å	128°	0.7 Å	**2.6 Å**	**28**	4.1 Å
1SSH	0.7 Å	C	8.4 Å	50°	1.1 Å	**3.2 Å**	**22**	4.2 Å
2C3I	0.2 Å	C	13.0 Å	70°	1.8 Å	**3.7 Å**	**299**	6.4 Å
1RXZ^c^	1.5 Å	β + C	8.9 Å	80°	1.9 Å	**4.3 Å**	**>500**	2.1 Å
2FMF	0.5 Å	α	16.1 Å	131°	0.4 Å	**5.0 Å**	**29**	3.5 Å
2P54	0.7 Å	α + C	13.0 Å	120°	2.2 Å	**9.7 Å**	**None**	8.1 Å
2FGR^c^	0.3 Å	C	7.3 Å	79°	1.6 Å	**10.1 Å**	**>500**	9.1 Å

Performance of peptide modeling onto Bound (A) and Unbound (B) protein receptor structures.

Legends as for Table 1.

#### Simulations on the bound receptor conformation (Bound docking)

We first assessed the performance of the modeling protocol on the bound backbone conformation of the receptor. Successful modeling within this setup validates our strategy for *sampling* peptide backbone conformations and rigid-body orientations, and our energetic *ranking* ability within the setting of an accurate receptor backbone structure. This setting is a prerequisite for more realistic and challenging simulations described below. The results of the bound docking benchmark are summarized in Table 1 and Fig. 2A. In all but one case (25/26; 96%), a near-native model of the interaction (as defined above) was sampled by our protocol, and in most cases (18/26; 69%), the near-native model was also ranked within the ten top-ranking clusters (top-ranked in 12/26; 46%). In half of the cases (12/26) the backbone atoms of the top-ranking modeled peptides were located within only one Ångstrom RMSD of the native peptide backbone. This is remarkable, as the peptide backbones in the benchmark adopt diverse secondary structures and backbone conformations, including unusual kinks, turns and coils that are particularly hard to predict *de-novo*: The starting backbone configurations include challenging cases where the initial peptide backbone isup to 17 Å away from the native conformation, and the RMSD of the initially extended peptide backbone from the native exceeds 125° in ϕ/ψ torsion space.

#### Robustness of the FlexPepDock ab-initio protocol to changes in starting conformation

**Figure 4:**
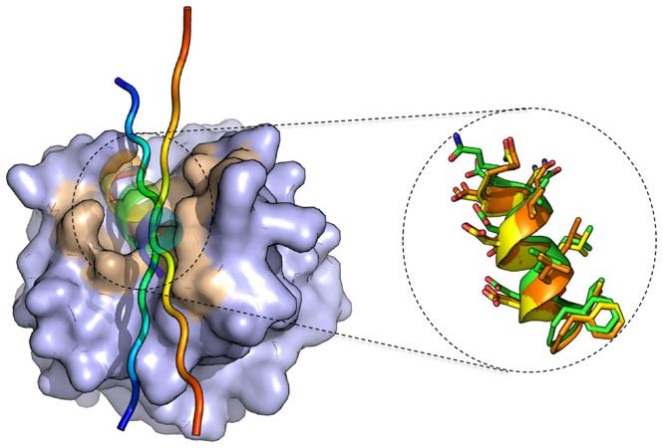
FlexPepDock *ab-initio* is robust to the initial starting position of the peptide, and therefore opens up the way towards fully blind peptide docking. The simulation of the structure of the CheZ-derived peptide bound to CheY (PDB-id 2FMF [55]) from two opposite starting orientations converge onto the same final conformation. (**A**) General view of the receptor structure (in gray; interface residues colored in light brown), the two initial extended peptide conformations (in cartoons), and the final helical conformation (in transparent cartoon). Peptide conformations are colored in rainbow. (**B**) Detailed atomic view of the top predictions from the two simulations (yellow and orange), highlighting the striking similarity of both predictions to the structure of the native peptide (green).

In our evaluation described above, we started from an extended peptide initially positioned at the correct binding site, by aligning one of the peptide residues to its native coordinates. Even though the peptide was free to change its orientation and was not constrained in any way during the simulation, it is important to validate the robustness of our docking protocol to this initial orientation. Therefore we reassessed performance by repeating the simulation starting from a different conformation (see Methods). The results indicate that our protocol is indeed robust to the precise initial orientation of the extended peptide at the binding site, since the results did not differ significantly among repeated runs (Table S3). Even in the case where two simulations that from peptides oriented in opposite directions, they converge onto one final structure of the peptide-protein complex (see Fig. 4), leading the way for fully blind peptide docking.

#### Comparison to peptide refinement protocol

The FlexPepDock *ab-initio* protocol uses our previously reported refinement protocol [26] as a sub-module, but significantly extends its scope thanks to a preceding low-resolution peptide *ab-initio* sampling step. In particular, the new protocol does not assume any prior information about the peptide backbone conformation, whereas the original refinement protocol works well mainly when the initial peptide conformation is within 5.5 Å Cartesian- and 50° torsion-space RMSD of the native conformation [26]. Indeed, the refinement protocol is able to sample a near-native conformation in only 10 of the 26 interactions (compared to 25/26 for theFlexPepDock *ab-initio* protocol described here; Fig. 2A), and these 10 are the easier cases where the native backbone conformation is already extended-like. Hence, the low-resolution stage of the *ab-initio* protocol is able to turn the harder cases into approximate models that can be refined to high-resolution.

#### Simulations on the free receptor conformation (Unbound docking)

In realistic scenarios, the bound receptor is obviously not part of the input when we try to dock a peptide to its protein receptor, and only the receptor in its free form can be used. The task of unbound docking is much more challenging, as the backbone conformation of the receptor protein may change upon binding, even though these changes are often very small for peptide-protein interactions [9]. We repeated the previous test for all cases where a structure of free receptor structure was available (Table S1). Again, in nearly all cases at least one near-native model was sampled by our protocol (12/14; 86%), and in half of the cases this model was ranked among the ten top-ranking clusters (7/14; 50%) (see Table 2; Fig. 2B and right panels in Fig. 3), indicating that the presented protocol is well suited for *de-novo* folding and docking in many practical settings.

### III. Partial success

Our protocol succeeded in modeling many of the complexes in our benchmarks, but also failed in some cases. Manual inspection of modeling failures revealed that in several of those, only part of the peptide has been modeled at high resolution, resulting in overall inaccurate models that do not meet the formal success criterion. In some cases, these partially accurate models may still be considered useful for practical applications. For instance, key motif residues may be modeled particularly well, (see our previous analysis in Raveh *et al* [26]), and other inaccuracies may be introduced by intrinsic motility of flexible peptide tails, and effects due to symmetry related contacts to the peptide that are not taken into account in our simulations (see below). Here we describe another case that involves *partial conformational changes of the receptor upon binding of the peptide*.

**Figure 5:**
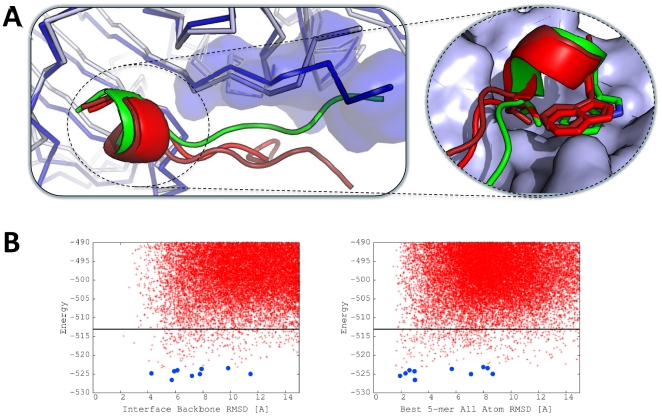
Redefining success. Partial accuracy due to a conformational change in the receptor in the interaction of the C-terminal region of FEN-1 with PCNA (PDB id 1RXZ [47]). (**A**) Structural view of this interaction in cartoon view: The free and bound conformations of the receptor are shown on the left in blue and gray ribbon, respectively. The native peptide is shown in green cartoon, and two of the top-scoring models are shown in red and pink. The C-terminal region of the receptor (highlighted) undergoes a conformational change upon binding to create a β-sheet pairing with the native peptide (green cartoon). Therefore, while the helical region of the peptide (residues 7–11, see inset) is well modeled in top-ranking models, the strand-part (residues 1–4) is not accurate. The inset on the right highlights the accurate recapitulation of certain atomic details in the helical peptide part. (**B**) Energy plots of this interaction: Left panel: The plot of the full peptide demonstrates that no near-native conformations are sampled or selected when considering the entire peptide sequence (peptide interface backbone RMSD, x-axis, *vs.* energy score, y-axis; same depiction as in Fig. 3). Right panel: The plot for the best pentamer substructure of the peptide demonstrates high accuracy, which can be attributed to the helical part of the peptide (note that this plot shows *all*atom RMSD).

The interaction of the C-terminal region of FEN-1 with PCNA involves a considerable conformational change that redefines part of the receptor peptide binding site: upon peptide binding, an intermolecular β-sheet interface is created between the two partners (contributing to overall 1.5 Å RMSD for interface Cα atoms between the bound and free receptor conformation). This conformational change has been suggested to explain how PCNA stimulates FEN-1 activity. In the structure of PCNA bound toa FEN-1 C-terminal peptide (PDB id 1RXZ [47]), the peptide consists of two parts: a β-strand (residues 1–4) connected to a short 3_10_ helix (residues 7–11; note that the 3_10_ helix is defined as a turn by STRIDE [48]). The β-strand forms the intermolecular β sheet, while the helix interacts with a region in the receptor that does not change upon binding. Not surprisingly, while we succeed in modeling the peptide conformation on the bound receptor conformation at high accuracy (Table 1), the models based on the free receptor conformation are only partly accurate (Table 2; Fig. 5): they describe the helical part at high accuracy, including most of the side-chain atoms (1.9 Å *all*-atom RMSD over the five C-terminal peptide residues for one of the top-10 models; Fig. 5), while the strand is not formed due to the lack of the corresponding partner strand in the receptor. This case demonstrates that even though the overall quality of the peptide model does not pass our ‘formal’ threshold for success, there is still a substructure that is modeled very accurately. Within the context of blind docking of the peptide conformation onto a free receptor conformation, this substructure could still serve as a useful starting point for subsequent peptide-based design and manipulation of the interaction.

### IV. Identification of new challenges

#### (1) Cases of accurate bound but inaccurate unbound docking highlight importance of receptor flexibility for peptide-protein interactions

While our protocol performs well in half of the unbound cases, examples like 1RXZ (described above and in Fig. 5) demonstrate the importance of including receptor flexibility in peptide docking, which is an ongoing work in our group. Even though the conformational changes of the receptor upon binding of peptides are usually fairly small (Table 2 and [9]), 5/14 peptide-protein complexes were modeled and ranked accurately only based on the bound receptor conformation, but not based on the free receptor conformation. These cases will be analyzed in more detail in a follow-up study and used to calibrate a protocol that includes receptor backbone flexibility.

#### (2) Cases of inaccurate bound docking might be due to crystal contacts that define the peptide conformation

We observed that in several structures (in particular where we fail in the simulations starting from the *bound* receptor structure), crystal symmetry operations reveal additional contacts to the peptide that are contributed by symmetry related molecules. These can represent a considerable fraction of the overall contacts that the peptide forms with its surrounding, and thus influence the structure that the peptide will adopt. Since we do not account for those contacts, our simulations might fail to accurately model, or select, peptide models that resemble the native crystal structure (e.g. we completely fail to identify the correct conformation for PDB-id 2J6F [49]). This intriguing finding suggests that solved peptide-protein complex structures might sometimes actually represent non-biological conformations. We are currently investigating in more detail how prevalent the influence of symmetry-related molecules on the peptide conformation is, and to what degree it affects our ability to identify and accurately model the key features in the peptide-protein interface of biological interactions.

#### (3) Towards blind peptide docking – integration with binding site prediction tools

We demonstrated that the present protocol is robust to the precise starting orientation of the peptide near the binding site (Fig. 4 and Table S3). Therefore, we foresee that it can be integrated with emerging techniques for identifying ligand and peptide binding sites, based on the chemical and the statistical features that characterize these sites (e.g. [9], [37], [50]). This would enable blind docking of peptides without any prior knowledge about either the binding site location or the peptide backbone conformation. We anticipate that such a tool will be applicable on a proteome-wide scale, and are working towards this direction.

### Conclusions

We have presented a Rosetta protocol to efficiently model the structure of a peptide bound to its receptor, using an optimization scheme that involves simultaneous full *ab initio* sampling of the peptide backbone conformation and its orientation on the receptor protein. This computationally intensive protocol samples a considerable conformation space, and consequently is able to identify near-native models within the top-ranking clusters for many challenging cases. These candidate structures provide an excellent starting point for the subsequent characterization and modulation of a peptide-mediated interaction: the atomic details of the interaction are revealed, and further refinement with the FlexPepDock refinement protocol described previously [26] can identify peptide residues that contribute significantly to binding affinity and specificity. This will significantly increase the number of peptide-mediated interactions that can be accurately characterized and manipulated.

## Supporting Information

Figure S1 **Energy landscape plots for models created by the FlexPepDock** ***ab initio*** **protocol.** Energy plots for Bound (left panel) and Unbound (right panel) docking runs are shown for the three successful and one failed simulations shown in Fig. 3 (see Table 1 for the full dataset). From top to bottom: 2O9V; 1N7F; 2A3I; and 2FGR. See Legend to Fig. 3 for more details.
(TIF)

Table S1 **The benchmark of peptide-protein interactions used in this study.**
(DOCX)

Table S2 **Number of successful predictions based on different scoring functions.** *Reweighted score* (blue) performs best. Results refer to detection of top-scoring models, prior to clustering (see Methods for more details).
(DOCX)

Table S3 **The FlexPepDock** ***ab-initio*** **protocol is robust to changes in starting conformation.** Similar results are obtained for two repeats of the protocol fromdistinct starting structures. The two starting structures are the extended conformation reported in Table 1 (in italics and parentheses), and an initial peptide orientation obtained by random translation and rotation of 3A and 30°, respectively).
(DOCX)
