# Supplementary figures and images for: Rosetta FlexPepDock ab-initio: Simultaneous Folding, Docking and Refinement of Peptides onto Their Receptors
Source: PLoS One. 2011 Apr 29;6(4):e18934. doi: 10.1371/journal.pone.0018934 (PMC3084719; doi:10.1371/journal.pone.0018934)

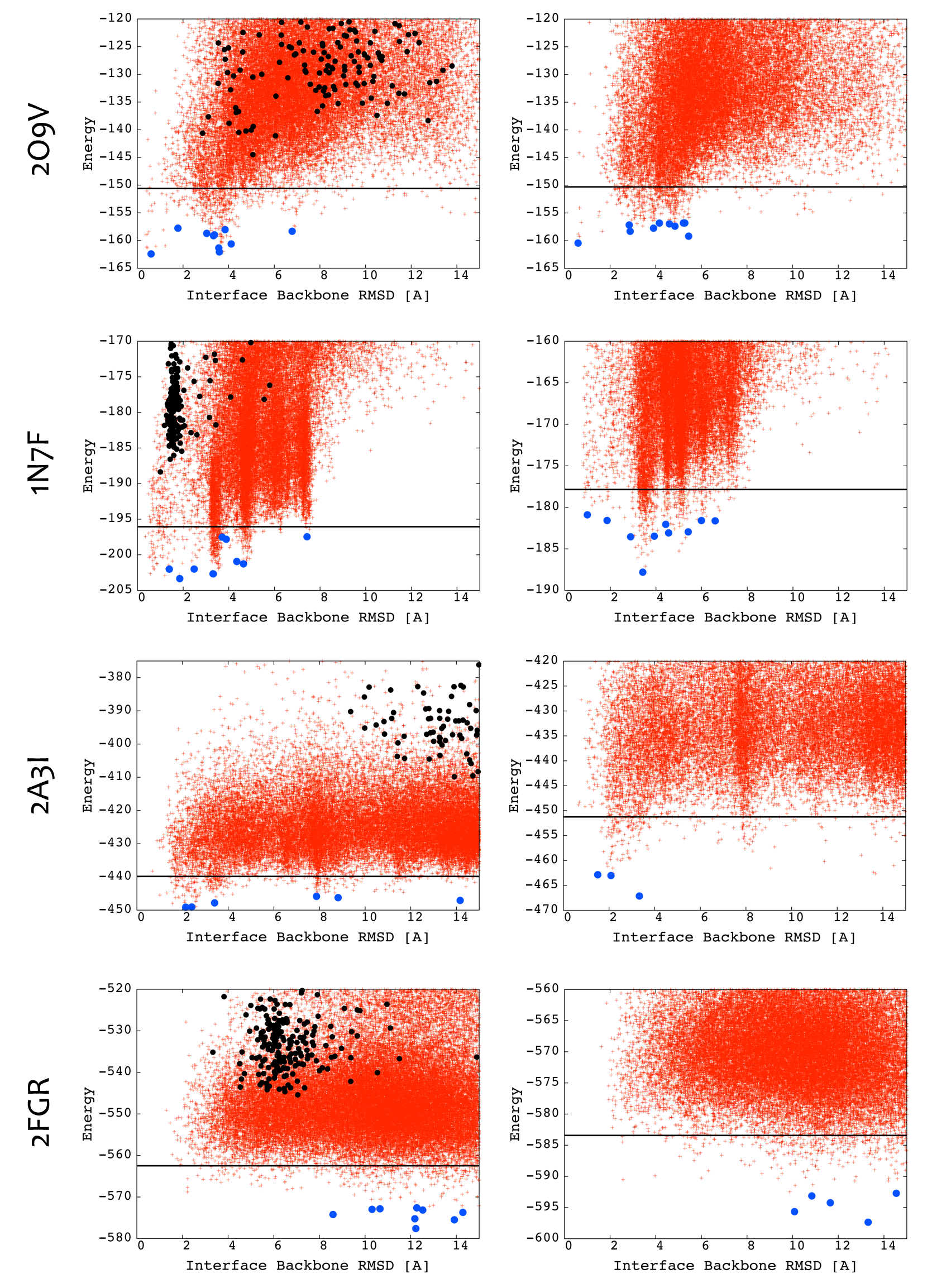

Supplement: Figure S1 — Energy landscape plots for models created by the FlexPepDock ab initio protocol. Energy plots for Bound (left panel) and Unbound (right panel) docking runs are shown for the three successful and one failed simulations shown in Fig. 3 (see Table 1 for the full dataset). From top to bottom: 2O9V; 1N7F; 2A3I; and 2FGR. See Legend to Fig. 3 for more details. (TIF) [file pone.0018934.s001.tif]
