# Supplementary material for: Rosetta FlexPepDock ab-initio: Simultaneous Folding, Docking and Refinement of Peptides onto Their Receptors
Source: PLoS One. 2011 Apr 29;6(4):e18934. doi: 10.1371/journal.pone.0018934 (PMC3084719; doi:10.1371/journal.pone.0018934)
Supplement: Table S1 — The benchmark of peptide-protein interactions used in this study. (DOCX) [file pone.0018934.s002.docx]

| (A) Bound  PDB id | Receptor protein chain | Peptide chain | Peptide secondary structure | Peptide length | Anchor residue (chosen randomly)‡ | Free Receptor protein PDB id | Unbound receptor Cα- iRMSD |
| --- | --- | --- | --- | --- | --- | --- | --- |
| 1AWR | C | I | C | 6 | 1 | 2ALF:A | 0.3 Å |
| 1ER8 | E | I | C | 8 | 5 |  |  |
| 1KL3 | C | G | α + C | 6 | 2 |  |  |
| 1N7F | B | D | β + C | 8 | 8 | 1N7E:A | 0.4 Å |
| 1NLN | A | B | β + C | 11 | 3 |  |  |
| 1NVR | A | B | β + C | 5 | 5 | 2QHN:A | 0.3 Å |
| 1QKZ | H | P | C | 10 | 5 |  |  |
| 1RXZ | A | B | β + C | 11 | 3 | 1RWZ:A | 1.5 Å |
| 1SSH | A | B | C | 11 | 9 | 1OOT:A | 0.7 Å |
| 1T7R | A | B | Α | 10 | 8 | 2AM9:A | 0.4 Å |
| 1TW6 | B | D | C | 6 | 4 |  |  |
| 1W9E | A | T | β + C | 5 | 4 | 1R6J:A | 0.6 Å |
| 1Z9O | AC | G | C | 9 | 7 |  |  |
| 2A3I | A | B | α + C | 12 | 2 | 2AA2:A | 0.3 Å |
| 2B1Z | B | D | Α | 9 | 5 |  |  |
| 2C3I | B | A | C | 8 | 1 | 2J2I:B | 0.2 Å |
| 2FGR | A | B | C | 8 | 1 | 2FGQ:X | 0.3 Å |
| 2FMF | A | B | Α | 13 | 1 | 1JBE:A | 0.5 Å |
| 2FNT | AB | P | C | 7 | 3 |  |  |
| 2J6F | A | C | C | 8 | 6 |  |  |
| 2O9V | A | B | C | 10 | 3 | 2O9S:A | 0.3 Å |
| 2P1K | A | C | β + C | 11 | 3 |  |  |
| 2P54 | A | B | α + C | 12 | 5 | 1I7G:A | 0.7 Å |
| 2R7G | C | D | α + C | 10 | 7 |  |  |
| 2VJ0 | A | P | C | 8 | 5 | 1B9K:A | 0.3 Å |
| 3D1E | A | P | C | 6 | 4 |  |  |

‡ Note that the index of the anchor residue is given relative to the first peptide residue in the peptide chain that was resolved in the crystal structure (not the residue index number in the original PDB structure).
