# Supplementary material for: Rosetta FlexPepDock ab-initio: Simultaneous Folding, Docking and Refinement of Peptides onto Their Receptors
Source: PLoS One. 2011 Apr 29;6(4):e18934. doi: 10.1371/journal.pone.0018934 (PMC3084719; doi:10.1371/journal.pone.0018934)
Supplement: Table S2 — Number of successful predictions based on different scoring functions. Reweighted score (blue) performs best. Results refer to detection of top-scoring models, prior to clustering (see Methods for more details). (DOCX) [file pone.0018934.s003.docx]

| (A) Bound docking | Score12*^a^* | Peptide score *^b^* | Interface score *^c^* | Reweighted score *^d^* |
| --- | --- | --- | --- | --- |
| top1 | 35% | 23% | 8% | **46%** |
| top-10 | 54% | 46% | 35% | **65%** |
| top-100 | 69% | 58% | 54% | **77%** |
| top-500 | 77% | 73% | 73% | **81%** |
| all samples | 96% | 96% | 96% | **96%** |

| (B) Unbound docking | Score12 | Peptide score | Interface score | Reweighted score |
| --- | --- | --- | --- | --- |
| top1 | 7% | 7% | 7% | **14%** |
| top-10 | 29% | 14% | 7% | **36%** |
| top-100 | 64% | 43% | 36% | **64%** |
| top-500 | 79% | 57% | 64% | **86%** |

*^a^ score12* – the default Rosetta full-atom energy function [38]

*^b^ peptide-score* – the contribution of the peptide residues to score12

*^c^* *interface-score* – the difference between the score of the complex and the score of each monomer (in analogy to ΔΔG of binding)

*^d^* *reweighted-score* – the sum of score12, peptide-score and interface-score
