# Supplementary material for: Rosetta FlexPepDock ab-initio: Simultaneous Folding, Docking and Refinement of Peptides onto Their Receptors
Source: PLoS One. 2011 Apr 29;6(4):e18934. doi: 10.1371/journal.pone.0018934 (PMC3084719; doi:10.1371/journal.pone.0018934)
Supplement: Table S3 — The FlexPepDock ab-initio protocol is robust to changes in starting conformation. Similar results are obtained for two repeats of the protocol fromdistinct starting structures. The two starting structures are the extended conformation reported in Table 1 (in italics and parentheses), and an initial peptide orientation obtained by random translation and rotation of 3A and 30°, respectively). (DOCX) [file pone.0018934.s004.docx]

| PDB id | Start-bb-RMSD | Best bb-iRMSD | Top10-ibb-RMSD |
| --- | --- | --- | --- |
| 1AWR | 6.7Å  *(5.3 Å)* | 0.8Å  *(0.8 Å)* | 0.8Å  *(0.9 Å)* |
| 1RXZ | 7.5Å  *(8.9 Å)* | 1.4Å  *(0.7 Å)* | 0.6Å  *(0.7 Å)* |
| 1T7R | 17.9Å  *(13.4 Å)* | 0.9Å  *(0.7 Å)* | 0.4Å  *(1.2 Å)* |
| 2A3I | 19.3Å  *(17.3 Å)* | 1.2Å  *(0.7 Å)* | 2.6Å  *(2.1 Å)* |
| 2O9V | 9.8Å  *(5.5 Å)* | 0.4Å  *(0.4 Å)* | 0.4Å  *(0.6 Å)* |
